# Assessment of influence of facemask treatment with skeletal anchorage on the temporomandibular joint using magnetic resonance imaging: a preliminary study

**DOI:** 10.1590/2177-6709.28.3.e2321302.oar

**Published:** 2023-07-24

**Authors:** Demet KAYA, Ilken KOCADERELI, Isil SAATCI

**Affiliations:** 1 Hacettepe University, Gün Hospital, Department of Oral and Dental Health Care, Orthodontics (Ankara, Turkey).; 2 Hacettepe University, Faculty of Dentistry, Department of Orthodontics (Ankara, Turkey).; 3 Hacettepe University, Faculty of Medicine, Department of Radiology (Ankara, Turkey).

**Keywords:** Class III malocclusion, Facemask, Magnetic resonance imaging, Skeletal anchorage, Temporomandibular joint

## Abstract

**Objective:**

The aim of the study was to investigate the influence of facemask treatment with skeletal anchorage on the temporomandibular joint (TMJ) using magnetic resonance imaging (MRI), in patients with Class III malocclusion, accompanied by maxillary retrusion.

**Methods:**

Fifteen patients with a mean age of 12.1±1.43 years were included in the study. All patients were treated using facemask with skeletal anchorage after eight weeks of Alternate Rapid Maxillary Expansion and Constriction (Alt-RAMEC) protocol. Magnetic resonance imaging was performed before and immediately after facemask treatment for TMJ evaluation. Disc position, condylar translation, degenerative changes of the condyles, and joint effusion were evaluated. To assess whether the alterations associated with the treatment were statistically significant, McNemar and marginal homogeneity tests were used.

**Results:**

After facemask treatment, a statistically significant change was observed in the disc position (an anterior disc displacement with/without reduction in five TMJs) (*p*<0.05). The alteration in the condylar translation was not statistically significant (*p*>0.05). This treatment did not cause degenerative changes of the condyles or effusion in any of the TMJs.

**Conclusion:**

Facemask treatment with skeletal anchorage following the Alt-RAMEC protocol had a minimal influence on the TMJ, only by means of disc position, which was not negligible. Long-term results of such treatment are required for following up the changes observed in the TMJs.

## RESUMO

**Objetivo:**

O objetivo desse estudo foi investigar a influência do tratamento com máscara facial com ancoragem esquelética na articulação temporomandibular (ATM), por meio de ressonância magnética (RM), em pacientes com má oclusão de Classe III acompanhada de retrusão maxilar.

**Métodos:**

Quinze pacientes com idade média de 12,1±1,43 anos foram incluídos no estudo. Todos os pacientes foram tratados com máscara facial com ancoragem esquelética após oito semanas de protocolo de Expansão Rápida da Maxila e Constrição Alternadas (Alt-RAMEC). Os exames de ressonância magnética foram realizados antes e imediatamente após o tratamento com máscara facial, para avaliação da ATM. Foram avaliados posição do disco, translação condilar, alterações degenerativas dos côndilos e derrame articular. Os testes de McNemar e de homogeneidade marginal foram utilizados para avaliar se as alterações associadas ao tratamento foram estatisticamente significativas.

**Resultados:**

Após o tratamento com máscara facial, uma mudança estatisticamente significativa foi observada na posição do disco (deslocamento anterior do disco com/sem redução em cinco ATMs) (p<0,05). A alteração na translação condilar não foi estatisticamente significativa (p>0,05). Esse tratamento não causou alterações degenerativas dos côndilos ou derrame em qualquer das ATMs.

**Conclusão:**

O tratamento com máscara facial com ancoragem esquelética ápós o protocolo Alt-RAMEC teve uma influência mínima na ATM, apenas quanto à posição do disco, que não foi desprezível. Resultados em longo prazo desse tratamento são necessários para acompanhar as mudanças observadas nas ATMs.

## INTRODUCTION

The purpose of the facemask treatment is to redirect or stimulate the growth of the maxilla forward, in patients with Class III malocclusion accompanied by maxillary retrusion. To increase its efficiency, the facemask has been applied in conjunction with rapid maxillary expansion (RME) and, recently, with the Alternate Rapid Maxillary Expansion and Constriction (Alt-RAMEC) protocol. However, some dental compensations (maxillary incisor proclination) were observed with these treatment protocols.^1^ ^,^ ^2^ Thus, a more rigid anchorage was used for a pure orthopedic forward movement of the maxilla, providing more stable results.^3^ Finally, facemask treatment with skeletal anchorage following the Alt-RAMEC protocol was applied to further increase the skeletal effect in severe cases and also to achieve skeletal effects for patients in the late treatment period.^4^

The conventional type of facemasks used for redirecting or stimulating the growth of the maxilla forward often obtains support from both the forehead and chin, and heavy forces are applied with these appliances for orthopedic effect. Grandori et al.^5^ reported that 75% of the force produced by the facemask is transmitted to the temporomandibular joint (TMJ). Any force transmitted to the TMJ may have an impact on TMJ components. In this situation, the risks of facemask treatment include posterior displacement of the condyle and anterior displacement of the articular disc, which may cause temporomandibular disorder (TMD); however, informations on this issue are controversial. Ricketts^6^ revealed that facemask treatment used for achieving a more normal association between the maxilla and mandible might promote TMD due to the force transmitted to the TMJ in the posterior direction. Contrarily, in a systematic review study recently published by Huang et al.,^7^ it was concluded that facemask treatment led to the displacement of the condyle, but presented evidence supporting the morphological adaptation of the TMJ to a changing functional status and that it might not be a risk factor for the development of TMD. 

The influence of facemask treatment on the TMJ has been evaluated using various methods, such as two-dimensional cephalogram, computed tomography (CT), cone beam computed tomography (CBCT), thin-plate spline analysis, mandibular position indicator, and Research Diagnostic Criteria for Temporomandibular Disorders.^8^ ^-^ ^14^ However, in the literature, a study assessing the influence of facemask treatment on the TMJ using magnetic resonance imaging (MRI) has not been conducted yet, except for a thesis study.^15^ It is well-known that MRI is the best imaging method that allows the examination of the soft tissues of the TMJ. Additionally, MRI has been shown to have a high accuracy rate in evaluating the osseous changes of the TMJ.^16^ Therefore, this study specifically aimed to investigate the MRI alterations in the TMJs of patients with skeletal Class III malocclusion accompanied by maxillary retrusion who underwent a facemask treatment with skeletal anchorage after the Alt-RAMEC protocol.

## MATERIAL AND METHODS

The present study was approved by the local ethics committee (approval number: LUT 06/91-20). Patients and their parents were informed about the treatment in detail, and written informed consent forms were obtained from the parents who agreed to participate in the study.

According to the result of power analysis, a sample size of 28 TMJs would achieve 81.377% power at a significance level of 0.050 using a one-sided non-inferiority test of correlated proportions when the standard proportion is 0.070. The maximum allowable difference between these proportions that still results in non-inferiority (the range of non-inferiority) is 0.120, and the actual difference of the proportions is 0.000.

**Figure 1:**
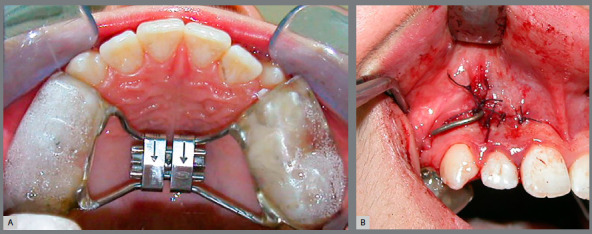
A) Intraoral photograph of bonded RME appliance used for Alt-RAMEC protocol. B) Intraoral photograph of the miniplate used for facemask application.

**Figure 2:**
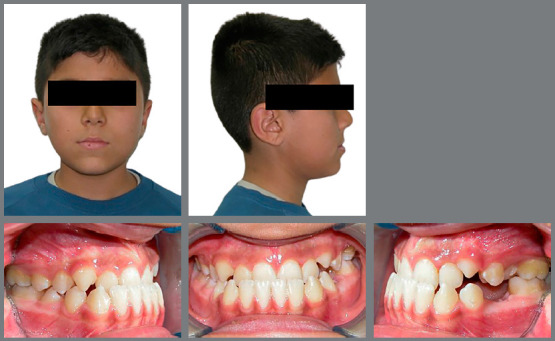
Extraoral and intraoral photographs of one patient before treatment.

**Figure 3:**
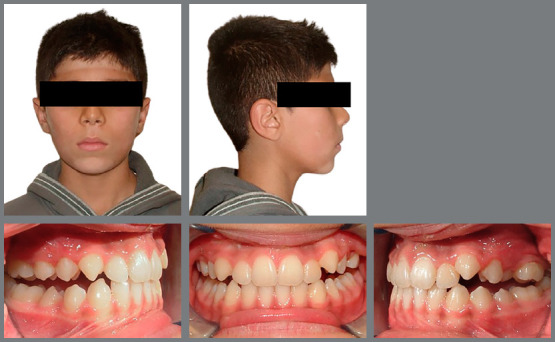
Extraoral and intraoral photographs of one patient after treatment.

Fifteen patients (9 girls, 6 boys) with a mean age of 12.1±1.43 years were included in this study. The inclusion criteria were as follows: (1) patients with no history of previous orthodontic or orthopedic treatment, (2) patients with no systemic diseases or congenital deformities, (3) patients with skeletal Class III malocclusion accompanied by maxillary retrusion (Wits appraisal of -2 mm or less), (4) patients with edge-to-edge or reverse incisor relationship, and (5) patients with no clinical symptoms of TMD such as joint sounds, limited mouth opening, mandibular shift, difficulty in chewing, and pain. Initial skeletal sagittal relationships of the patients in terms of ANB angle and Wits appraisal were -1.3±1.76° and -7.1±3.09mm, respectively. All patients were treated with Delaire-type facemask with miniplate anchorage (Multipurpose Implant; Tasarimmed, İstanbul, Turkey) bilaterally inserted on the lateral nasal wall of the maxilla, following eight weeks of Alt-RAMEC protocol with bonded RME appliance (Fig 1). Alt-RAMEC protocol began with expansion, followed by final constriction (considering that maxillary expansion was not required). The time for each expansion or constriction course was two weeks, and the daily activation of the screw for each course was 0.5 mm a day. The miniplates were inserted immediately after Alt-RAMEC protocol. After soft tissue healing, 100 g of force per side with a direction of 30° forward and downward to the occlusal plane was applied via elastics between the miniplates and facemask. Subsequently, the force in the same direction was increased by 350-400 g per side at the second week of the facemask treatment. Patients were instructed to wear the facemask full time, except for meals. When the desired movement of the maxilla was obtained for a good profile, the facemask treatment was finished. Initial and final photographs of one of the patients included in the study are presented in Figures 2 and 3. The total treatment time including the Alt-RAMEC protocol was 9.9±2.63 months. 

To evaluate the alterations in the TMJs, MRI (1.5-Tesla MRI unit; Siemens Symphony, Erlangen, Germany) was performed before Alt-RAMEC protocol and immediately after facemask treatment in all patients. Images were acquired at both closed and opened mouth positions. TMJs were imaged both in the sagittal and coronal planes. Sagittal sections were acquired perpendicular to the long axis of the condyle, and coronal sections were acquired parallel to the long axis of the condyle. To prevent muscle fatigue at opened mouth position during imaging, an acrylic bite block was placed at a thickness of 10 mm below the maximum interincisal opening, and patients were instructed to be in a resting position while contacting the anterior teeth with the bite block.

TMJs were evaluated in terms of disc position, whether normal or displaced. Moreover, disc displacement, whether with or without reduction, was also assessed. TMJs were also evaluated for condylar translation during mouth opening: whether normal, restricted, or excessive. The condylar translation was considered normal if the condyle drifted until or just beyond the posterior slope of the articular eminence; restricted, if the condyle was behind the articular eminence; and excessive, if the condyle drifted beyond the articular eminence (hypermobility).^17^ Additionally, the presence or absence of degenerative changes of the condyles and joint effusion were assessed. All MRI examinations were performed by one researcher. Following the initial reporting of the MRIs, the MRI examinations were evaluated by the same researcher again.

The Statistical Package for the Social Sciences v. 23.0 software (International Business Machines Corp.; Armonk, NY, USA) was used for data analysis. To assess whether the alterations in TMJs associated with the treatment were statistically significant, McNemar and marginal homogeneity tests were used. A*p*-value less than 0.05 was determined as statistically significant.

## RESULTS

With this treatment approach, improvement was observed in skeletal sagittal relationship of the patients (ANB angle, 1.5±1.67°; Wits appraisal, -2.9±2.80 mm).

At the beginning of the facemask treatment, out of the 30 TMJs, 28 had bilateral normal disc positions, and 2 TMJs (in one patient) had an anterior disc displacement with reduction. Condylar translation was normal in all TMJs except for 1 TMJ, which the condyle was behind the articular eminence during mouth opening. None of the condyles had degenerative changes. Effusion was observed only in 2 TMJs of the patient having a bilateral anterior disc displacement with reduction. 

**Figure 4:**
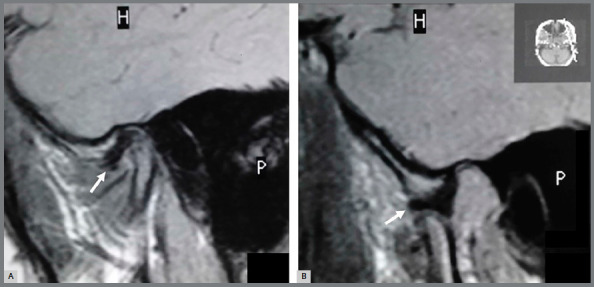
Anterior disc displacement with reduction (arrow). Oblique sagittal magnetic resonance images in closed mouth (**A**) and opened mouth (**B**) positions.

**Figure 5:**
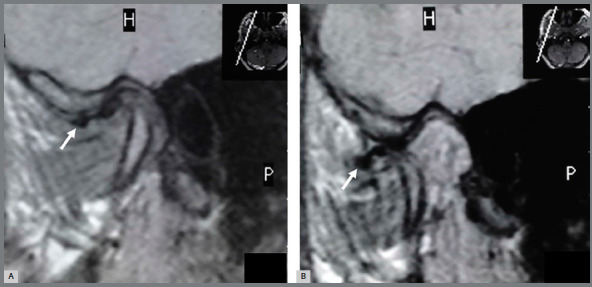
Anterior disc displacement without reduction (arrow). Oblique sagittal magnetic resonance images in closed mouth (**A**) and opened mouth (**B**) positions.

After the facemask treatment, a statistically significant change was observed in the disc position (*p*<0.05). The disc positions remained normal in 23 of the 30 TMJs, whereas an anterior disc displacement with reduction was observed in 4 TMJs (bilateral in one patient, unilateral in two patients) and an anterior disc displacement without reduction in 1 TMJ (in another patient) (Figs 4 and 5). Two TMJs continued to have an anterior disc displacement with reduction. In 28 TMJs, normal condylar translation remained stable, and the preexisting restricted condylar translation in another TMJ improved. However, a restricted condylar translation after the facemask treatment was observed in 1 TMJ. The alteration in the condylar translation was not statistically significant (*p*>0.05). No degenerative changes were detected in any of the condyles. Moreover, this treatment did not cause effusion in any of the TMJs, and the severity of preexisting joint effusion in one patient did not change (Table 1).

**Table 1:** Alterations in temporomandibular joints of patients treated with facemask with skeletal anchorage following the Alternate Rapid Maxillary Expansion and Constriction (Alt-RAMEC) protocol.

	Disc position (after treatment)					
	Normal	Anterior disc displacement with reduction	Anterior disc displacement without reduction	Total	Total (n)	p-value
Disc position (before treatment)	Normal	23	4	1	28	0.034*^,a^
	Anterior disc displacement with reduction	0	2	0	2	
	Anterior disc displacement without reduction	0	0	0	0	
	Total (n)	23	6	1	30	
Condylar translation (after treatment)						
		Normal	Restricted	Excessive	Total (n)	p-value
Condylar translation (before treatment)	Normal	28	1	0	29	1.000^b^
	Restricted	1	0	0	1	
	Excessive	0	0	0	0	
	Total (n)	29	1	0	30	
Degenerative changes of the condyle (after treatment)						
		Absent	Present	Total (n)	p-value	
Degenerative changes of the condyle (before treatment)	Absent	30	0	30	1.000	
	Present	0	0	0		
	Total (n)	30	0	30		
Joint effusion (after treatment)						
		Absent	Present	Total (n)	p-value	
Joint effusion (before treatment)	Absent	28	0	28	1.000^b^	
	Present	0	2	2		
	Total (n)	28	2	30		

n = number; *p<0.05.

^a^ Marginal homogeneity test; ^b^McNemar test.

## DISCUSSION

In the literature, the skeletal and dental effects of facemask treatment have been well documented. However, a limited number of studies regarding the influence of this treatment on TMJ exists. Thus, this study aimed to increase the knowledge about the influence of facemask on the TMJs of patients with Class III malocclusion and maxillary retrusion during the growth and development period. None of the patients included in this study were in the early treatment period, which was a disadvantage in terms of highly interdigitated sutures. To loosen circummaxillary sutures, at least 12-15 mm of expansion is needed,^18^ resulting in the maxilla to be wider than the mandible. Therefore, the Alt-RAMEC protocol was applied in all patients to facilitate the forward movement of the maxilla without overexpansion by weakening the circummaxillary sutures before facemask application. Additionally, a skeletal anchorage was used to achieve a pure orthopedic forward movement of the maxilla, by transmitting the orthopedic force directly to the circummaxillary sutures.

The impact of facemask treatment on the TMJ has been evaluated clinically and radiologically.^8^ ^,^ ^9^ ^,^ ^12^ ^,^ ^14^ ^,^ ^15^ Although clinical evaluation helps in establishing the diagnosis of TMD, it may not provide sufficient information for the overall diagnosis. Accordingly, the clinical evaluation of the TMJ should be supported with radiological examination. In facemask studies, researchers often used two-dimensional cephalograms for the radiological evaluation of the TMJ.^8^ ^,^ ^10^ ^,^ ^14^ Subsequently, they preferred more advanced methods such as CT and CBCT for a more detailed evaluation.^7^ ^,^ ^9^ To date, a study assessing the alterations in TMJs using MRIs in patients with Class III malocclusion treated via a conventional facemask or a facemask with skeletal anchorage following the Alt-RAMEC protocol has not been conducted yet. MRI is a relatively reliable method used to assess the TMJ. It was reported that the accuracy rate of MRI was 95% in evaluating the position of the disc and the soft tissue around it. Moreover, MRI has been reported to be 93% accurate in evaluating the osseous alterations of the TMJ.^16^ Since it has no radiation side effects, it is considered superior compared to other advanced imaging methods such as CT and CBCT. This advantage of MRI is specifically important for children whose growth and development period continue. However, MRI is expensive for routine clinical use.

In the present study, only patients with no clinical symptoms of TMD were included, but MRI examinations of 2 TMJs (in one patient) indicated an anterior disc displacement with reduction before the facemask treatment. This finding was consistent with the findings of a previous study showing that an anterior disc displacement could be detected in the radiological examination of symmetric or asymmetric patients with Class III malocclusion and without clinical symptoms of TMJ .^19^ There could be some reasons for this situation. First, a disc displacement with reduction can remain asymptomatic for a long time, due to the adaptive physiological processes that may occur. The primary adaptive physiological process is the retrodiscal fibrosis, which can explain why the patients having disc displacement with reduction feel no pain. Another reason could be the change in the morphology of the condylar head resulting from remodeling. In addition, it is possible that the neo-neuromuscular system will balance out the desired occlusion, keeping the condyle in its physiologic position, and prompting accomplishment of a normal disc position. The same 2 TMJs also had a bilateral joint effusion. Another TMJ had a unilateral restricted condylar translation. Despite the studies showing that there might be an association between TMD and Class III malocclusion,^20^ ^,^ ^21^ TMD was not detected in most of the TMJs in the present study, except for these 3 TMJs. 

Potential causes of TMJ alterations after facemask treatment include the force produced by the facemask, forward movement of the maxilla, posterior displacement of the condyle, and growth. Among these, the force produced by the facemask is the primary factor. A large part of this force is transmitted to the TMJ.^5^ It was reported that the stress levels created on the TMJ by orthopedic forces were smaller than those during normal clenching and chewing functions and therefore would not damage the TMJ. ^22,23^ Even so, its effect on the TMJ was investigated by several researchers because facemask was hypothesized to create a different vector in the TMJ from the vector occurring in the TMJ during normal functions and has a potential to alter the position of the condyle posteriorly. Posterior displacement of the condyle may result in anterior displacement of the disc. In the present study, the positions of the discs did not change during mouth opening/closing in most of the TMJs (83.3%) after maxillary protraction. This was consistent with the result of the study showing that orthopedic treatment with the appliances getting support from the chin did not affect the position of the disc in patients with Class III malocclusion.^24^ However, in the present study, disc displacements in post-treatment MRIs was diagnosed in 16.7% of TMJs. The rate of disc displacement in the present study was higher than the rates observed in some studies.^12^ ^,^ ^14^ However, these studies used only clinical examination for the diagnosis, which may be insufficient to establish the overall diagnosis when compared to radiological examination. Similarly, Köse^15^, in his doctoral thesis, found that the disc was slightly displaced in the anterior direction after the application of orthopedic facemask. Since in the present study MRI examination was performed immediately after the facemask treatment, it was not known whether the disc displacements in 5 TMJs would be permanent or not. After that, more clear findings could be provided by retrieving and reevaluating the records. Additionally, it is possible for the articular structures to adapt morphologically to the new functional state in children, due to continued growth and development. 

The limit of condylar movement in the posteroanterior direction during function is from the center of the glenoid fossa to the apex or slightly anterior to the articular eminence. Dynamic movements of the condyle can be assessed with MRI. To the best of our knowledge, this study is the first to evaluate the condylar translation before and after facemask treatment with skeletal anchorage following the Alt-RAMEC protocol. In the present study, it was observed that the restricted translation of 1 condyle improved with facemask treatment, but a condyle with preexisting normal translation had a restricted movement after facemask treatment. This may be caused by a real restriction in condylar translation, or by the patient not fully opening the mouth. 

Applying force to the TMJ using the Delaire-type facemask leads to compressive movement of the condyle through the glenoid fossa in the posterior direction.^11^ As a result, a degenerative change on the condyle can be expected. In animal studies, after retraction forces, it was shown that the remodeling process of the condyle was altered, and a resorption was observed at the posterior surface of the condyle.^25^ With facemask treatment, no degenerative changes of the condyles were detected in the present study. The absence of degeneration on the condyle can be attributed to the condyle’s morphological adaptation mechanism, as patients continue to grow and develop, and the condyle is still under modification and significantly varies.^26^ It was also demonstrated that the increase in occlusal vertical dimension obtained by the installation of dental appliances promoted the thickness of condylar cartilage.^27^ In addition to growth, the application of the facemask over bonded RME appliance may be another factor compensating for the resorption that may occur on the condyle.

TMJ effusion is a condition characterized by an excessive collection of intra-articular synovial fluid that can be easily diagnosed on MRI examination. No effusion was detected in 95% of patients having normal disc position.^28^ However, it could be frequently observed in asymptomatic or symptomatic patients with TMD. ^29,30^ In this study, effusion was not observed in any of the TMJs having normal/abnormal disc position, either before or after treatment, except for 2 TMJs of 1 patient. It has been reported that the severity of effusion increases in patients with anterior disc displacement.^31^ The aforesaid 2 TMJs of 1 patient also had a bilateral anterior disc displacement with reduction. Although an orthopedic force was applied to the TMJs, the severity of effusion in this patient did not increase. In fact, discussions regarding the association between anterior disc displacement and joint effusion are still ongoing, and no definitive conclusion has yet been reached because joint effusion was not diagnosed in some of the patients having anterior disc displacement.^30^ The absence of joint effusion in 5 TMJs having an anterior disc displacement after maxillary protraction is also a new contribution to this discussion. In the literature, there is no information concerning the TMJ effusion in patients treated with facemask. With the present study, a new knowledge is considered to be added to the literature.

The present study has some limitations. First, it would be better if the facemask group could be compared with an untreated control group having Class III malocclusion resulting from maxillary retrusion, but it was not possible due to ethical reasons. Another problem was the small sample size. A larger sample size may improve the precision of the results. Additionally, further studies with long-term results of such treatment are required for following up the results observed in the TMJs of the patients with Class III malocclusion. Nonetheless, the present evaluation provided an important information about the influence of facemask treatment with skeletal anchorage following the Alt-RAMEC protocol on the TMJ. 

## CONCLUSION

Considering the results of the present study, the following conclusions can be made:

» Facemask treatment with skeletal anchorage following the Alt-RAMEC protocol had a minimal influence on the TMJ only regarding disc position, which was not negligible.
» With the exception of 1 condyle having restricted translation, the condylar translation was normal in most of TMJs after the facemask treatment.
» This treatment approach did not cause degenerative changes of the condyles or effusion in any of the TMJs.
» Long-term results of such treatment are required for following up the changes observed in the TMJs.
